# Modulation of Glutamatergic Burst Activity by Hydrolysed Arabinoxylan Rice Bran: A Multielectrode Array Study in Human-Induced Pluripotent Stem Cell-Derived Neurones and Astrocytes

**DOI:** 10.7759/cureus.77694

**Published:** 2025-01-20

**Authors:** Basant K Puri, Cecilia Catuogno-Cal, Ivan Verduci

**Affiliations:** 1 Molecular Biology, Cambridge Advanced Research, Cambridge, GBR; 2 Psychology, Neapolis University Pafos, Pafos, CYP; 3 Electrophysiology, Cambridge Advanced Research, Cambridge, GBR; 4 Electrophysiology, 3Brain AG, Genova, ITA

**Keywords:** astrocyte, biobran, glutamate, glutamatergic neurone, induced pluripotent stem cell

## Abstract

The natural product MGN-3 (Biobran) is a defatted, partially hydrolysed rice bran-derived hemicellulose enzymatically modified with an extract of *Lentinus edodes*. It has a high proportion of arabinoxylan. It has a protective action against intracerebroventricular streptozotocin-induced murine sporadic Alzheimer’s disease and reverses spatial memory deficit in this disease model. The aim was to test the hypothesis that MGN-3 increases glutamatergic burst activity in human neuronal and glial cells by conducting an in vitro multielectrode array-based micro-electrophysiological study in a cultured mixture of human glutamatergic neurones, GABAergic neurones and astrocytes. The effects of MGN-3 at two concentrations, 0.750 g L^-1^ and 0.375 g L^-1^, and vehicle (control), on glutamatergic burst activity in a triculture of human-induced pluripotent stem cell (hiPSC)-derived GABAergic neurones, glutamatergic neurones and astrocytes were studied. The change in the number of glutamatergic bursts normalised to the vehicle control was analysed using a normal or Gaussian generalised linear model. This statistical model was highly significant (*p* = 1.468 × 10^-17^). Both MGN-3 concentrations were associated with highly significant main effects. These results provide strong evidence to reject the null hypothesis that MGN-3 does not affect glutamatergic burst activity in human neuronal and astrocytic cells. The study's strengths include the novel use of hiPSC-derived neurones and astrocytes and the robust statistical significance of the findings. Limitations include in vitro conditions that may not fully replicate in vivo conditions, potential variability in hiPSC-derived cell preparations, and the need to test other neuronal subtypes or additional doses to assess dose-dependent effects. These should be addressed in future studies.

## Introduction

The natural product MGN-3, also known as Biobran, Lentin Plus, BRM4 and Ribraxx, is a defatted, partially hydrolysed rice bran-derived hemicellulose enzymatically modified with an extract of *Lentinus edodes*, having a high proportion of arabinoxylan [1,2]. In a murine model, Ghoneum and El Sayed [3] have recently demonstrated a protective action of MGN-3 against intracerebroventricular streptozotocin-induced sporadic Alzheimer’s disease; MGN-3 reversed spatial memory deficit in this disease model. Spatial memory deficit is an early predictor of Alzheimer’s disease; indeed, spatial navigation deficits may be a marker of pre-clinical Alzheimer’s disease [4,5].

The electrophysiological balance between excitatory and inhibitory neurotransmission is altered in Alzheimer’s disease, with changes in the cross-talk between neurones and glial cells being important [6]. The presynaptic arm involves glutamatergic and GABAergic neurones, in association with astrocytes, while the postsynaptic arm again involves glutamatergic and GABAergic neurones, this time in association with microglia [6]. Several lines of evidence point to the importance of glutamate in this cerebral homeostatic network. First, it has been shown that inhibition of central astrocytic glutamate uptake with dihydrokainic acid in rats is associated with impaired spatial memory [7]. Second, mammalian spatial memory retrieval is regulated by glutamatergic neurones in the postrhinal cortex [4]. Furthermore, spatial memory retrieval entails the transmission of spatial information in the postrhinal cortex-ventrolateral orbitofrontal cortex pathway; this pathway is glutamatergic [4]. Spatial memory regulation involves synchronisation between the supramammillary nucleus and the dentate gyrus (which it innervates), with this synchronisation involving supramammillary nuclear glutamate release [8]. An enhanced glutamate response in the dentate gyrus occurs during active avoidance learning [9]. Inhibition of this hippocampal dentate gyral glutamate response is associated with impaired spatial learning and memory, while reversal of this inhibition allows recovery of spatial learning [10]. Finally, while it is well-established that human methamphetamine addiction is associated with memory deficits [11], in a murine model of methamphetamine withdrawal-disrupted spatial memory it has been shown that restoration of hippocampal dorsal CA1 astrocytic glutamine clearance rescues spatial memory [12]. Taken together, these studies support the existence of a strong link between memory function and the glutamatergic network.

Glutamatergic N-methyl-D-aspartate receptor (NMDAR)-dependent long-term potentiation (LTP) is strongly implicated in the mediation of learning and memory [13,14]. Indeed, in the seminal work of Collingridge and colleagues, published over 40 years ago, the ionophoretic application of N-methyl-DL-aspartate to rat CA1 hippocampal neurones was found to be associated with glutamatergic burst activity [15]. It is now established that the CA1 synaptic long-term plasticity which mediates LTP can be stimulated by theta-burst glutamatergic activity at both postsynaptic NMDARs and α-amino-3-hydroxy-5-methyl-4-isoxazole propionic acid receptors (AMPARs); such glutamatergic burst activity is associated with NMDAR calcium ion influx [14,16]. The association of theta-burst activity and mnemonic function is not confined to the hippocampus but appears to occur elsewhere in a hippocampal-diencephalic-cortical network [17].

Based on the above studies, it was hypothesised that MGN-3 increases glutamatergic burst activity in human neuronal and glial cells. The aim of this study was to test this hypothesis by conducting an in vitro multielectrode array (MEA)-based micro-electrophysiological study in a cultured mixture of the human neurones and glial cells associated with a cerebral homeostatic network implicated in Alzheimer’s disease [6], namely glutamatergic and GABAergic neurones, together with astrocytes. Microglia were omitted from this mixture for one practical reason, namely that burst activity would not be measurable from them, and for one theoretical reason, namely that although microglia are part of the postsynaptic arm of the homeostatic model of Alzheimer’s disease, they do not feature in the hippocampal-diencephalic-cortical network which subserves memory and learning.

## Materials and methods

Experimental design

The MGN-3 was tested at two concentrations and also at zero concentration. The zero-concentration consisted of the "vehicle" in which the non-zero MGN-3 preparations were diluted, but without any MGN-3. Thus, the vehicle alone acted as a control group. The effects of MGN-3, at the two concentrations, and vehicle (control group), on glutamatergic burst activity in a triculture of human induced pluripotent stem cell-derived GABAergic neurones, glutamatergic neurones and astrocytes were studied. The study took place at Cambridge Advanced Research, Cambridge, UK.

MEA plate preparation

Six-well MEA plates (CorePlate™; 3Brain AG, Pfäffikon, Switzerland) were used for all experiments. One day prior to plating cells, sterile filtered 0.1% polyethyleneimine (Thermo Fisher Scientific Inc., Waltham, USA) in 1 × borate buffer (Thermo Scientific) was added to each well and left for one hour, rinsed twice with sterile phosphate-buffered saline, twice with sterile water, and allowed to dry overnight in a sterile microbiological safety cabinet.

Cell culture and maintenance

Cells were cultured in BrainPhys™ neuronal medium (STEMCELL Technologies Inc., Vancouver, Canada) supplemented with 100 g/mL laminin (natural, murine; Gibco, Thermo Fisher Scientific Inc.), 70 micrograms/mL gentamicin (Gibco), 1 × iCell Neural Supplement B (FUJIFILM Cellular Dynamics, Inc. (FCDI), Madison, USA), 1 × iCell Nervous System Supplement (FUJIFILM Cellular Dynamics) and 1 × N-2 Supplement (Gibco).

Cryopreserved human induced pluripotent stem cell-derived GABAergic neurones (iCell GABANeurons; FUJIFILM Cellular Dynamics), glutamatergic neurones (iCell GlutaNeurons; FUJIFILM Cellular Dynamics) and astrocytes (iCell Astrocytes 2.0; FUJIFILM Cellular Dynamics) were thawed in a 37°C water bath for 150 seconds and added dropwise to 1 mL medium in a 50-mL tube while swirling to prevent osmotic shock. An additional 2 mL (astrocytes, glutamatergic neurones) or 6 mL (GABAergic neurones) of medium was added. A total of 20 microlitres of cell solution was removed for cell counting. The cell suspensions were centrifuged for five minutes at 400 g. The supernatant was discarded and the cells were resuspended in medium to a total volume of 1 mL. Cells were manually counted independently by two researchers using a haemocytometer.

Neurones and astrocytes were pre-mixed into a triculture and seeded directly onto the plate microchips in 70 microlitre droplets containing 78,000 glutamatergic neurones (56%), 42,000 GABAergic neurones (30%) and 20,000 astrocytes (14%). The plates were placed in a humidified incubator at 37°C and 5% CO_2_ for one hour, after which 300 microlitres of medium was added. A further 1 mL of medium was added 90 minutes later. Cells were maintained with half changes of the medium every two to three days.

MEA recordings

**Figure 1:**
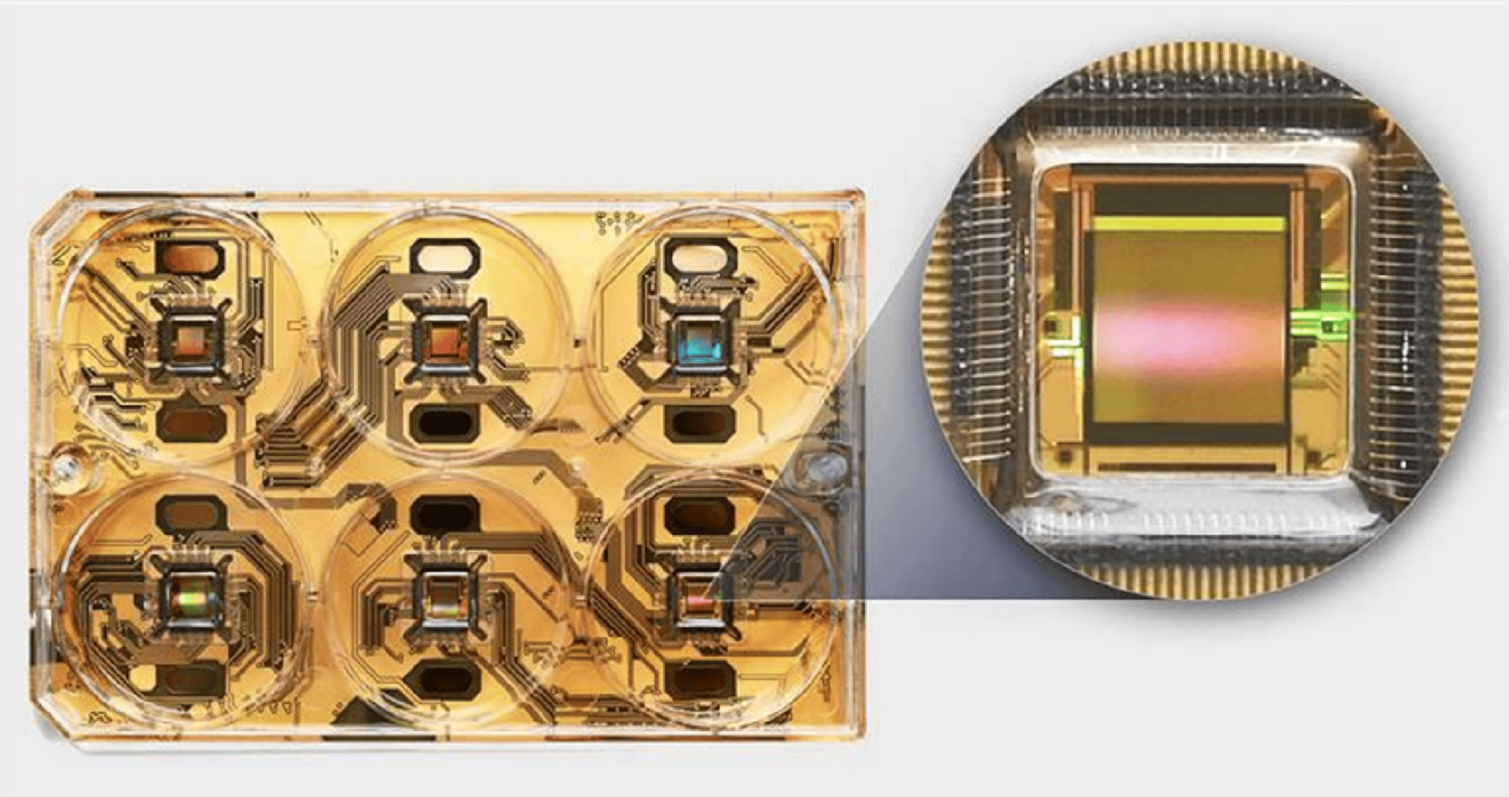
The 3Brain CorePlate™ six-well culture plate was used to record electrical activity from cell cultures, with 2304 electrodes recording simultaneously in each well.

All recordings were acquired with the HyperCam MEA system (3Brain) which maintains a constant environment of 37°C and 5% CO_2_. Six-well plates were used and configured with 2304 electrodes per well (electrode pitch 60 micrometres, electrode dimensions 25 micrometres × 25 micrometres, total recording area 2.9 mm × 2.9 mm) (see Figure 1).

MEA plates were allowed to equilibrate on the HyperCam for 10 minutes prior to each recording. On the 22nd day in vitro (DIV 22), baseline spontaneous activity was recorded for five minutes prior to compound addition, after which 10 micromolar picrotoxin (Thermo Scientific) was added to induce syncope-like epileptic seizures. After five to seven minutes, test compounds were added manually to each well and 15-minute recordings were taken immediately after.

Traces were sampled at 10 kHz, high-pass filtered at 100 Hz and acquired with noise blanking compression (3.5 standard deviations (SD)/4 SD low/high threshold).

Compound preparation and treatment

MGN-3 (Daiwa Pharmaceuticals, Tokyo) was solubilised in ethanol to form a stock solution of a concentration of 0.375 g/mL. The MGN-3 was diluted at 1:500 and 1:1000, corresponding to concentrations of 0.750 g/L and 0.375 g/L, respectively, directly into the culture medium. Two wells per MEA plate were reserved for vehicle controls (0.1% and 0.2% ethanol by volume). Picrotoxin, MGN-3 and vehicle controls were added by pipetting directly into the wells with the plate remaining in the HyperCam. To ensure consistency across wells, a total volume of 20 microlitres was added to each well, with medium making up the remaining volume after the addition of the compound or vehicle.

Spike and network analysis

All detection analyses were carried out using BrainWave 5.0 (3Brain). Spikes were detected using a hard threshold of -40 mV with artificial intelligence validation. Wells with fewer than 30 active units (<0.05 spikes/min) were discarded from analyses; a total of four wells out of 24 were discarded. Bursts were recorded when at least three spikes were detected on the same electrode with inter-spike intervals of less than or equal to 100 ms. The parameters of the algorithm were tailored to each recording. All spike and burst results were checked manually, to ensure agreement with visual assessment, by two researchers.

Raw data were extracted from the software and manually processed. The effect of the MGN-3 was analysed by comparing the five-minute baseline recording to the last five minutes of the immediate post-dose recording and the five-minute one-day post-dose recording.

Statistical analysis

The statistical tests conducted were as follows. First, Gaussian generalised linear modelling. For this modelling, the response variable was the change in the number of glutamatergic bursts normalised to the vehicle control. Since the neurones form a synchronised network in which the activity on any one electrode is strongly related to the activity on any other active electrode, the data were grouped well. Hence, the normalised glutamatergic burst variable was analysed using a normal or Gaussian generalised linear model, with the compound (vehicle and two concentrations of MGN-3), time (baseline and two follow-up times) and well as factors, with the identity canonical link function. Second, the arithmetic mean number of bursts in each of the three groups was calculated, together with the corresponding standard error and 95% confidence interval. These statistical analyses were carried out using R v. 4.2.1 (The R Foundation for Statistical Computing, Vienna, Austria) and JASP 0.17.2.1 (The JASP Team, Amsterdam, Netherlands) [18,19], as was the graphical plotting.

## Results

**Figure 2:**
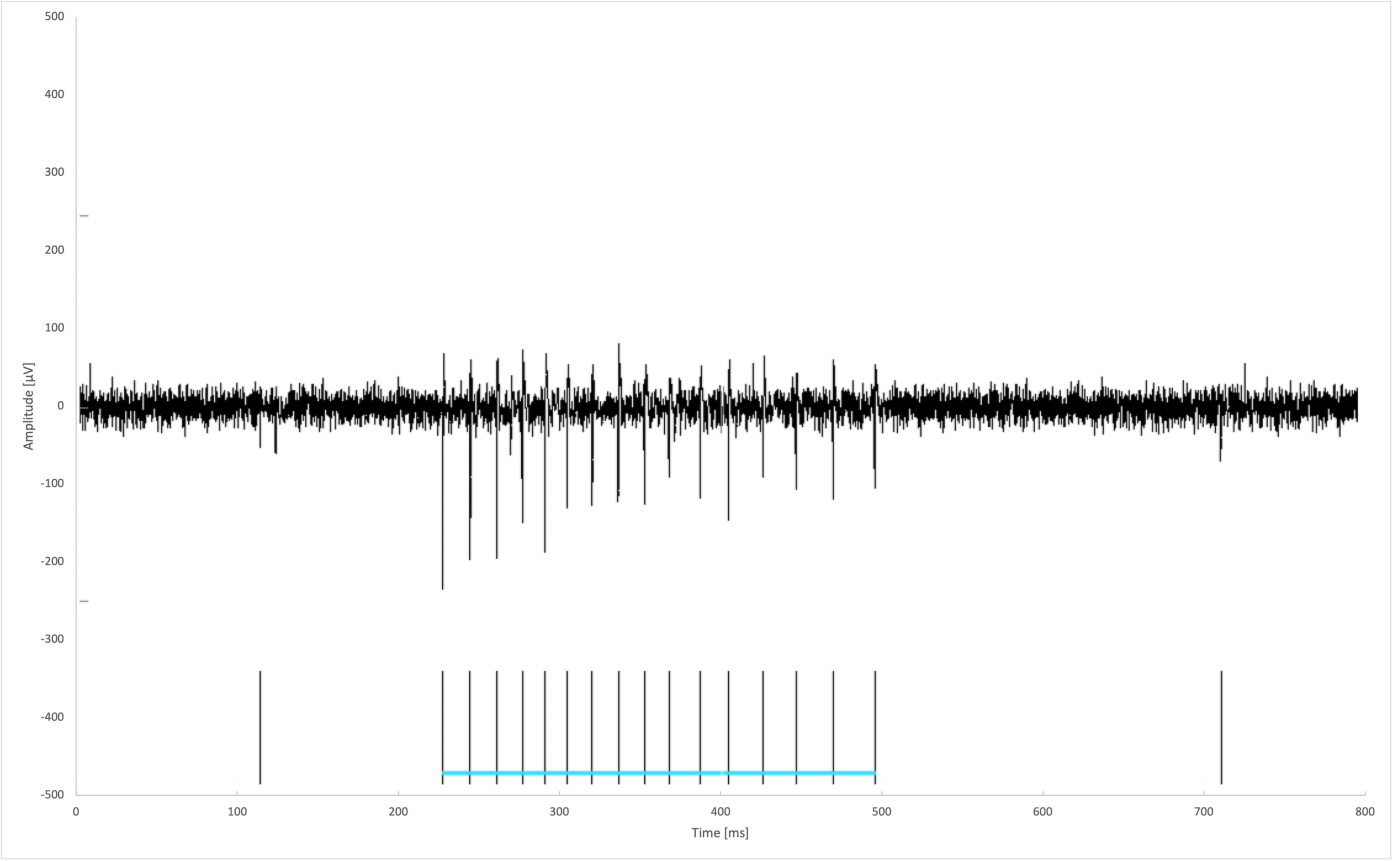
Example trace from an electrode showing a characteristic bursting pattern. The horizontal blue line groups together the spikes attributed to a single burst.

A characteristic bursting pattern, together with the spikes attributed to a single glutamatergic burst, is shown in Figure 2.

**Figure 3:**
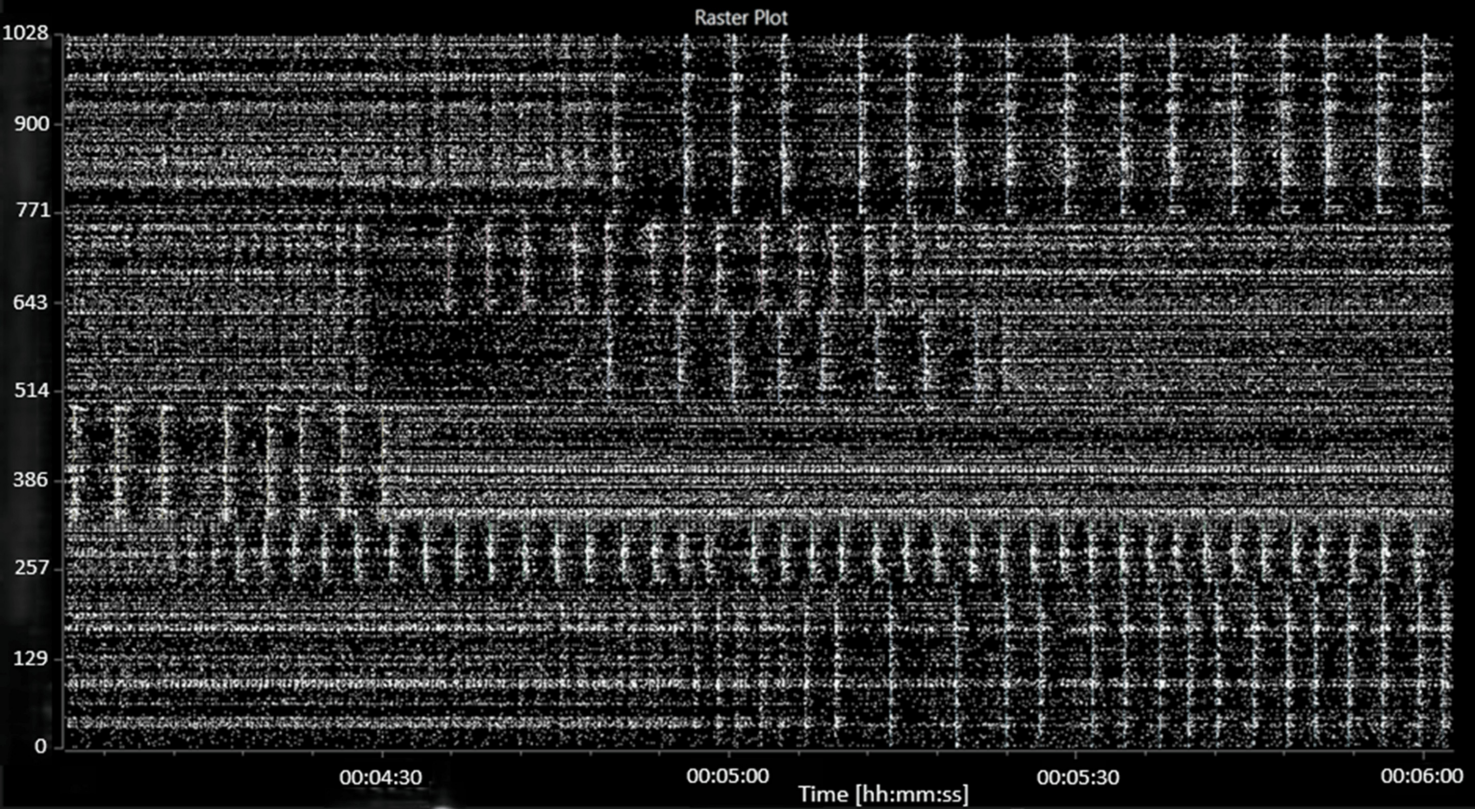
A raster plot showing characteristic baseline electrical activity (before the addition of picrotoxin or MGN-3). The abscissa (x-axis) shows the time, in hours:minutes:seconds. The ordinate (y-axis) shows the electrode number.

Spontaneous electrical activity was recorded in all wells. For subsequent analyses, wells with fewer than 30 active units (<0.05 spikes per second) were discarded. During the baseline recording, spontaneous spiking, bursting and network bursting activity were recorded from all wells. The baseline activity was characterised by an average mean (standard error) firing rate (MFR) of 140 (12.2) spikes per second per well, an average burst frequency of 9.3 (1.3) bursts per second per well and an average of 73 (4) active bursting units per well. The network bursting activity was intermittent with sections of asynchronous activity and sections of regular network bursting (see Figure 3).

**Figure 4:**
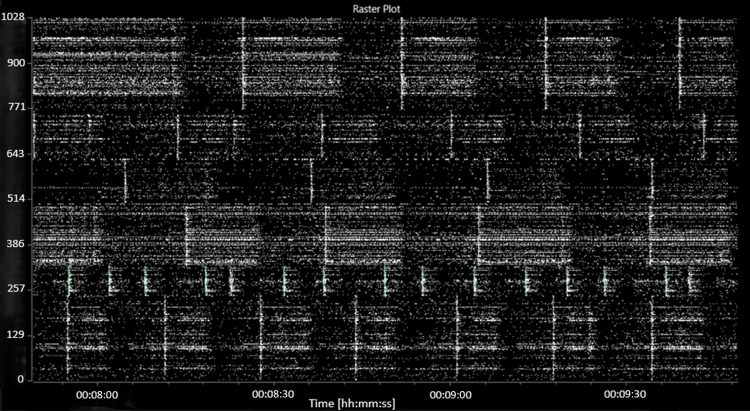
Raster plot showing characteristic activity after the addition of picrotoxin. The abscissa (x-axis) shows the time, in hours:minutes:seconds. The ordinate (y-axis) shows the electrode number.

The addition of picrotoxin caused a consistent decrease in both the average MFR and the average burst frequency to 62 (8.4) spikes per second per well and 4.3 (1.0) bursts per second per well, respectively. A decrease in the number of active bursting units was also noted, to 50 (5) active bursting units per well. An increase in network bursting frequency and synchronicity was observed. A raster plot of characteristic activity after the addition of picrotoxin is shown in Figure 4.

**Figure 5:**
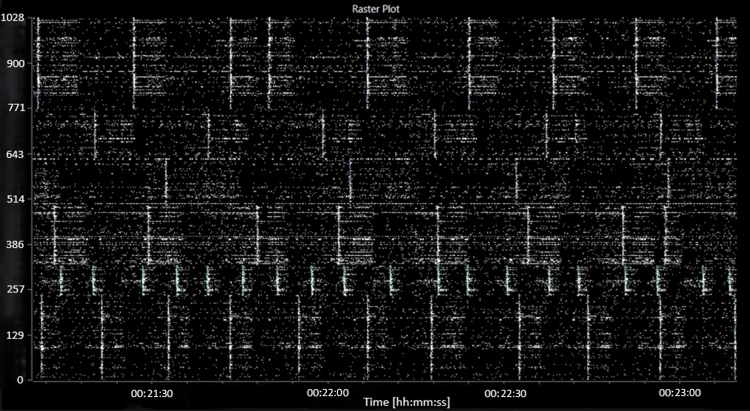
Raster plot showing characteristic activity after the addition of MGN-3 (following the addition of picrotoxin). The abscissa (x-axis) shows the time, in hours:minutes:seconds. The ordinate (y-axis) shows the electrode number.

The addition of MGN-3 caused a further decrease in the average MFR to 57 (6) spikes per second per well and a strong recovery in the number of active bursting units back up to 70 (4) spikes per second per well. A slight increase in the burst frequency to 4.6 (0.6) bursts per second per well was seen. No significant changes were seen to the network activity, which broadly resembled the picrotoxin-induced activity. A raster plot of characteristic activity after the addition of MGN-3 is shown in Figure 5.

**Figure 6:**
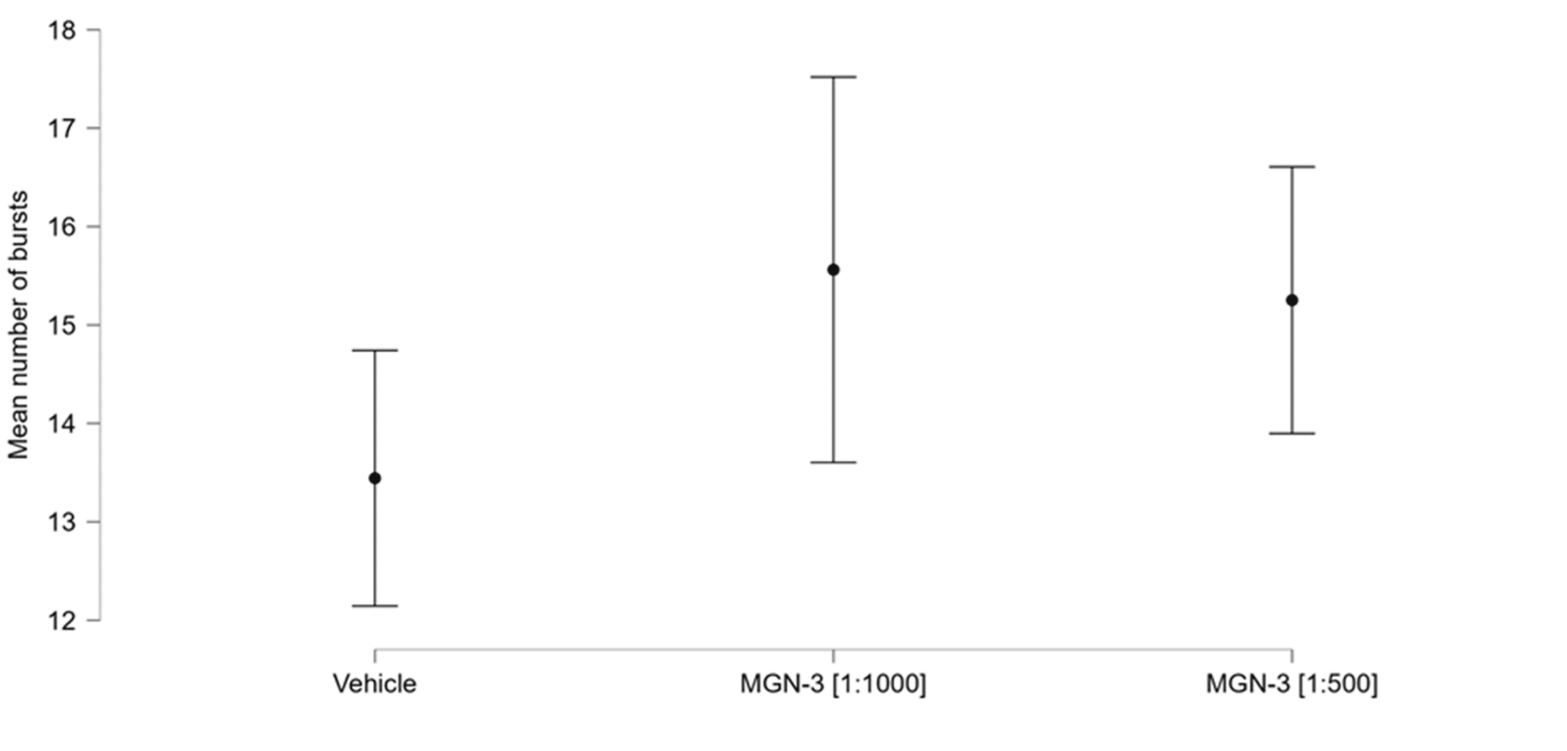
Group mean bursts and 95% confidence intervals.

The mean burst number for MGN-3 at the 1:1000 concentration was 15.561 (0.999; five wells; 1634 electrodes); for MGN-3 at the 1:500 concentration this was 15.252 (0.692; eight wells; 2892 electrodes); while that of the vehicle was 13.443 (0.662; seven wells; 2924 electrodes). Figure 6 shows a plot of these group means and their corresponding 95% confidence intervals.

The statistical model was highly significant (chi-squared (40) = 19.969, *p* = 1.468 × 10^-17^). Both concentrations of MGN-3 showed highly significant effects, as shown in Table 1. Nine of the wells (three corresponding to the lower MGN-3 concentration and six to the higher MGN-3 concentration) also showed significant effects; these are not shown in Table 1. None of the vehicle wells showed a significant effect; nor was the intercept of the model significant. Time was not significant. The use of the Difference in Fits (DFFITS) statistic showed that there was no evidence of any unduly influential observations.

**Table 1:** Gaussian generalised linear modelling coefficients.

	Estimate	Standard error	t	p
MGN-3 (1 in 1000)	-0.684	0.156	-4.389	8.103 × 10^-5^
MGN-3 (1 in 500)	1.291	0.156	8.278	3.330 × 10^-10^

## Discussion

The highly significant main effects of both concentrations of the compound provide strong evidence to reject the null hypothesis. These results support the hypothesis that MGN-3 increases glutamatergic burst activity in human neuronal and astrocytic cells. A majority of the compound wells were also associated with significant main effects, while none of the vehicle wells showed a significant effect, which is not unexpected given that these were controls.

An alternative explanation of the results could be that MGN-3 induced syncope-like epileptic seizures by acting on chloride channels of the GABAergic neurones. However, this is highly unlikely for two reasons. First, the cells were pre-treated with picrotoxin, which blocks the GABA_A_ receptor chloride channel ion flow activated by GABA [20]. Second, there is no evidence that MGN-3 acts as a competitive antagonist of picrotoxin; indeed, the structure of a polysaccharide composed of activated hemicellulose and β1,3-glucan does not readily suggest such an action [21].

It could be argued that pre-treatment with picrotoxin meant that the experiments were performed under hyper-excitatory conditions which do not have a relation to the Alzheimer’s disease phenotype. However, several groups have argued that hyper-excitatory (or excitotoxic) conditions are related to Alzheimer’s disease [22-24]. Indeed, picrotoxin pretreatment was employed for this reason in an Alzheimer’s disease-related in vitro study of the potential of taurine to prevent the neurotoxicity of β-amyloid and glutamate receptor agonists [24]. Nevertheless, if, as the present results indicate, MGN-3 evokes an increase in glutamatergic activity, then this should be clearly, and likely even better, visible in cells which have not undergone picrotoxin pre-stimulation; such a future study is therefore indicated.

It is premature to argue that the present results suggest that MGN-3 is likely to have a therapeutic role in Alzheimer's disease (including early in this disease). On the one hand, the results harmonise with the finding of a protective effect of MGN-3 in a sporadic Alzheimer's disease murine model [3]. On the other hand, to date, there is no published evidence of a beneficial role of this nutritional supplement in respect of human Alzheimer's disease. A randomised, placebo-controlled pilot study in adult humans with cognitive decline would be in order.

Regarding the strengths of the study, these include the novel use of hiPSC-derived neurones and astrocytes and the robust statistical significance of the findings. In terms of the limitations of the study, one issue relates to the pre-treatment with picrotoxin. This has been discussed above. Also, in vitro conditions may not fully replicate in vivo conditions. Other potential limitations include potential variability in hiPSC-derived cell preparations and the need to test other neuronal subtypes or additional doses to assess dose-dependent effects. These should be addressed in future studies. Another issue is the fact that the study was carried out on a single layer of neurones and astrocytes. This is clearly different from the three-dimensional geometry existing in the mammalian brain. Three-dimensional high-resolution functional imaging, using micro-needle electrodes, has very recently become available. These micro-needle electrodes permit access to electrical activity from within three-dimensional neuroglial arrays without causing cellular damage. It would be appropriate to use such new technology in future in vitro follow-up studies.

## Conclusions

This study supports the hypothesis that MGN-3 increases glutamatergic bursts in human neurones and astrocytes. Further studies are required to help clarify the following. First, the potential mechanism by which MGN-3 enhances glutamatergic activity (for example, through receptor modulation or metabolic pathways). Second, how the present findings might translate into in vivo models or clinical settings. Third, the relevance of specific concentrations used in relation to therapeutic doses. Indeed, a more detailed dose-response in vitro study, without picrotoxin pre-treatment, would be appropriate, to establish the optimum dosage.
